# Local Information as an Essential Factor for Quantum Entanglement

**DOI:** 10.3390/e23060728

**Published:** 2021-06-08

**Authors:** Zhaofeng Su

**Affiliations:** 1 LINKE Lab, School of Computer Science and Technology, University of Science and Technology of China, Hefei 230027, China; zfsu@ustc.edu.cn; Tel.: +86-15156070489; 2 Key Laboratory of Wireless-Optical Communications, University of Science and Technology of China, Chinese Academy of Sciences, Hefei 230027, China

**Keywords:** quantum entanglement, geometric parameters, two-qubit system

## Abstract

Quantum entanglement is not only a fundamental concept in quantum mechanics but also a special resource for many important quantum information processing tasks. An intuitive way to understand quantum entanglement is to analyze its geometric parameters which include local parameters and correlation parameters. The correlation parameters have been extensively studied while the role of local parameters have not been drawn attention. In this paper, we investigate the question how local parameters of a two-qubit system affect quantum entanglement in both quantitative and qualitative perspective. Firstly, we find that the concurrence, a measure of quantum entanglement, of a general two-qubit state is bounded by the norms of local vectors and correlations matrix. Then, we derive a sufficient condition for a two-qubit being separable in perspective of local parameters. Finally, we find that different local parameters could make a state with fixed correlation matrix separable, entangled or even more qualitatively entangled than the one with vanished local parameters.

## 1. Introduction

Entanglement is a fundamental concept in quantum mechanics, which was firstly recognized by Einstein, Podolsky and Rosen (EPR) [1] and named by Schrödinger [2] in 1935. Since then, quantum entanglement has been extensively studied and it has been widely accepted that entanglement is one of the most basic characteristics of quantum mechanics [3]. In recent year, the rising of quantum computation and quantum information starts the second revolution of quantum technology [4]. Entanglement is not only of theoretical significance in quantum mechanics but also plays an indispensable role in quantum computation and quantum information. In the last decades, many novel protocols for quantum information processing tasks have been proposed, which are applications of quantum entanglement. For example, this includes quantum key distribution [5], teleportation [6], quantum dense coding [7], and quantum repeaters [8,9].

In a composite quantum system, there exist quantum states which cannot be interpreted as ensembles of product states. This feature of quantum mechanics is known as quantum entanglement. A quantum state which has this feature is said to be entangled. Otherwise, the state is separable. Formally, the state $\rho_{AB}$ of a bipartite quantum system A⊗B is separable if it can be decomposed into the form as follows,

(1) $$\rho_{AB}=\sum_{k}p_{k}\rho_{k}^{A}⊗\rho_{k}^{B},$$

where $\sum_{k}p_{k}=1$ with each $p_{k}\geq 0$, $\rho_{k}^{A}$ and $\rho_{k}^{B}$ are density operators on quantum systems *A* and *B*, respectively. Otherwise, $\rho_{AB}$ is entangled. The problem of detecting the entanglement of a state is also known as separability problem. The classical determination of separability problem for a general quantum state has been proved to be NP-hard [10].

The simplest quantum state that can exhibit quantum entanglement is the state of a two-qubit system. The general density operator $\rho_{AB}$ of a two-qubit quantum system can be represented by the combination of the identity operator and the generators of the SU(2) algebra [11] as follows,

(2) $$\rho_{AB}=\frac{1}{4}\left(I⊗I+\vec{r}\cdot \vec{\sigma }⊗I+I⊗\vec{s}\cdot \vec{\sigma }+\sum_{j,k=1}^{3}T_{jk}\sigma_{j}⊗\sigma_{k}\right),$$

where $\vec{\sigma }=\left(\sigma_{1},\sigma_{2},\sigma_{3}\right)$ is the vector of Pauli matrices, $\vec{r}$ and $\vec{s}$ are vectors in $R^{3}$ with norm less than or equal to 1, and the coefficients $T_{jk}=tr\left(\sigma_{j}⊗\sigma_{k}\rho_{AB}\right)$ compose a 3×3 real matrix *T*. Equation (2) is considered as the geometric representation of quantum state which is known as the Bloch representation [12]. The Bloch representation can be generalized to high dimensional and multi-party quantum systems [11].

In the last few decades, a variety of operational approaches and geometric approaches are investigated to reveal the separability of quantum systems. The first progress for separability problem was made by Clauser et al. in 1969 [13]. They proposed the well known CHSH inequality, the violation of which is a sufficient evidence of quantum entanglement. Horodecki et al. derived an analytical result for the violation of CHSH inequality. They proved that the general density operator of a two-qubit system in Equation (2) violates the CHSH inequality if and only if the sum of the largest two eigenvalues of the matrix $TT^{T}$ is greater than 1 [14]. The Horodeckis’ further found that the upper bound of trace norm ${∥T∥}_{tr}\leq 1$ must hold for any separable state of a two-qubit system [15]. Several necessary conditions for the separable states of more complex quantum systems have been derived [16,17,18,19], which are reduced to the Horodeckis’ result in the case of two-qubit system.

In perspective of operational approach, Peres found that the partial transposition of density operators are necessarily positive if they are separable [20]. This criterion for separability problem is known as positive partial transposition (PPT). It has been proved that PPT is a sufficient and necessary condition for the separability of 2×2 and 2×3 quantum systems [21]. Attempts have been made to generalize the PPT criterion to more complex scenarios where the quantum systems are of higher dimensions or multiple participators are involved [22,23,24,25]. The entanglement of a bipartite quantum system can be quantified by the von Neumann entropy of either of the two subsystems, which is also known as entanglement of formation [26]. The entanglement of formation of a two-qubit system is analytically related to the corresponding concurrence which can act as another measure in its own right [27,28]. Our numerical analysis shows that the PPT criterion and concurrence of a general two-qubit quantum system are qualitatively equivalent for detecting entanglement.

Consider marginal systems of the two-qubit composite system in Equation (2). The density operator for the single qubit of system *A* is $\rho_{A}=tr_{B}\left(\rho_{AB}\right)=\frac{1}{2}\left(I+\vec{r}\cdot \vec{\sigma }\right)$. For the system *B* is $\rho_{B}=tr_{A}\left(\rho_{AB}\right)=\frac{1}{2}\left(I+\vec{s}\cdot \vec{\sigma }\right)$. Obviously, the properties of the marginal systems are completely described by the vectors $\vec{r}$ and $\vec{s}$, which are known as local parameters of the joint quantum system, respectively. Moreover, it is acknowledged that the matrix *T* contains all the information about the correlations between the two subsystems A and B [16]. Through numerical analysis, however, we found that there are a large amount of entangled two-qubit states beyond the capability of the aforementioned analytical conditions which only consider the correlation matrix *T*. Our study shows that the local parameters have unignorable effects on the entanglement. The case where local parameters vanishes has been completely investigated by Horodeckis [15]. However, there are few literatures that focus on the cases where local parameters perform an indispensable role.

In this paper, we investigate the effects of local parameters on the entanglement of a two-qubit system. We find a upper bound and a lower bound for concurrence in geometric parameters. We derive a sufficient condition for being separable in geometric parameters based on the PPT criterion. By investigating a special case with general diagonal correlation matrix and nonvanished local vectors, we find that the entanglement of a two-qubit system is heavily affected by local parameters. For a state with fixed correlation parameters, the different local parameters could make the state separable, entangled or even more qualitatively entangled than the state with vanished local parameters.

The paper is organized as follows. In Section 2, we introduce the tools for qualitatively detecting and quantitatively measuring quantum entanglement of a two-qubit system. In Section 3, we systematically analyze the simplification of geometric representation of a general two-qubit state via local unitary operations. In Section 4, we analyze the upper bound and lower bound for concurrence in geometric parameters. In Section 5, we exploit the effect of local parameters on entanglement in both perspective of qualitative analysis and quantitative measures.

## 2. The Qualitative and Quantitative Analysis of Quantum Entanglement

In this section, we introduce the PPT criterion and concurrence of two-qubit states.

### 2.1. Qualitative Detection of Quantum Entanglement

The aforementioned PPT criterion is a sufficient and necessary condition for detecting separability of 2×2 and 2×3 quantum systems. Suppose $\rho_{AB}=\sum_{ijkl}\lambda_{ijkl}\left|i\rangle \langle j\right|⊗\left|k\rangle \langle l\right|$ is the density operator of a general two-qubit system expressed in standard basis. The partial transposition of the second subsystem of the density operator $\rho_{AB}$ is $\rho_{AB}^{PT}=\sum_{ijkl}\lambda_{ijkl}\left|i\rangle \langle j\right|⊗(|k\rangle {\langle l|)}^{T}=\sum_{ijkl}\lambda_{ijkl}\left|i\rangle \langle j\right|⊗\left|l\rangle \langle k\right|$. Peres found that is the partial transposition of either subsystem, say $\rho_{AB}^{PT}$, is necessary positive if $\rho_{AB}$ is a separable state [20].

### 2.2. Quantitative Measure of Quantum Entanglement

A widely accepted measure of quantum entanglement is known as entanglement of formation, which is denoted as E(·) for any bipartite quantum state in this paper. For a general bipartite state $\rho_{AB}$, its entanglement of formation is defined as follows [26],

(3) $$E\left(\rho_{AB}\right)\equiv \min\sum_{k}p_{k}E\left(\psi_{k}\right),$$

where the minimum is over any possible ensemble $\left\{p_{k},|\psi_{k}\rangle \right\}$ such that $\rho_{AB}=\sum_{k}p_{k}|\psi_{k}\rangle \langle \psi_{k}|$ and $E\left(\psi_{k}\right)\equiv S\left(tr_{B}(|\psi_{k}\rangle \langle \psi_{k}|)\right)=S\left(tr_{A}(|\psi_{k}\rangle \langle \psi_{k}|)\right)$ is the Von Neumann entropy of either subsystem of the joint state $|\psi_{k}\rangle$.

Hill and Wootters found an exact formula of E(·) for a general two-qubit quantum system as follows,

(4) $$E\left(\cdot \right)=h\left(\frac{1+\sqrt{1-C{\left(\cdot \right)}^{2}}}{2}\right),$$

where h(·) is the binary entropy of a real number between 0 and 1 and C(·) stands for the concurrence of a general two-qubit quantum system. Clearly, concurrence can act as a measure of quantum entanglement for two-qubit quantum system in its own right.

For a general pure state |ψ〉, the corresponding concurrence is $C\left(\psi \right)=|\langle \psi |\tilde{\psi }\rangle |$ where $|\tilde{\psi }\rangle =\left(Y⊗Y\right)|\psi^{*}\rangle$ and $|\psi^{*}\rangle$ is the complex conjugate of |ψ〉 [27]. For a general two-qubit mixed state ρ, Wootters found the corresponding concurrence as follows [28],

(5) $$C\left(\rho \right)=\max\left\{0,\lambda_{1}-\lambda_{2}-\lambda_{3}-\lambda_{4}\right\},$$

where $\lambda_{k}$ are eigenvalues of the operator $\sqrt{\sqrt{\rho }\tilde{\rho }\sqrt{\rho }}$ in the decreasing order and $\tilde{\rho }=\left(Y⊗Y\right)\rho^{*}\left(Y⊗Y\right)$. Wootters derived this analytical result by showing the existence of a decomposition $\rho =\sum_{i}q_{i}|z_{i}\rangle \langle z_{i}|$ such that $q_{i}\geq 0$ and $C\left(z_{i}\right)=C\left(\rho \right)$ for every pure state $|z_{i}\rangle$. We have reconcluded this fact as a lemma in a recent publication [29].

## 3. The Equivalent Simplification of Geometric Representation

We consider Equation (2) as geometric representation of a general two-qubit system, which contains 15 real variables. The analysis of separability problem is very complex because of massive variables. It has been showed that local unitary operations could not affect the separability of joint quantum system [3]. Thus, it is possible to reduce the number of coefficients in the geometric representation of the quantum system by applying local unitary operations. In this section, we systematically discuss the simplification of geometric representations in Equation (2) via local unitary operations.

### 3.1. Simplified Geometric Representation in Generalized Basis

Recall that a set of operators $\left\{\mu_{j}\right\}$ on an inner product space *V* forms a basis of operators on the space if $tr\left(\mu_{j}\mu_{k}^{†}\right)=\lambda_{jk}\delta_{jk}$ for some non-zero parameters $\lambda_{jk}$. Note that the identity operator *I* and Pauli matrices forms a basis of the space of a single qubit system. In Lemma 1, we find that any density operator of a two-qubit system can be represented in some basis such that the corresponding correlation matrix is diagonal.

**Lemma** **1.** *Suppose $\rho_{AB}$ is the general density operator of a two-qubit system. Then, there are two sets of operators $\left\{\sigma_{k}^{\prime }\right\}$ and $\left\{\sigma_{k}^{\prime \prime }\right\}$ such that the density operator $\rho_{AB}$ can be presented as follows,*

(6) $$\rho_{AB}=\frac{1}{4}\left(I⊗I+\vec{r^{\prime }}\cdot \vec{\sigma^{\prime }}⊗I+I⊗\vec{s^{\prime }}\cdot \vec{\sigma^{\prime \prime }}+\sum_{m=1}^{3}d_{m}\sigma_{m}^{\prime }⊗\sigma_{m}^{\prime \prime }\right),$$

*where the correlation matrix is diagonal. The operators $\left\{\sigma_{k}^{\prime }\right\}$ and $\left\{\sigma_{k}^{\prime \prime }\right\}$ plus the identity operator can compose two bases of the operators on single qubit system, respectively.*

**Proof.**  Recall that any density operator $\rho_{AB}$ can be represented as the form in Equation (2). Suppose the singular value decomposition of the real matrix *T* is $T=SDV^{T}$ where $D=diag(d_{1},d_{2},d_{3})$ is diagonal matrix, *S* and *V* are real orthogonal matrices. Let $\sigma_{m}^{\prime }\equiv \sum_{j=1}^{3}s_{jm}\sigma_{j}$ and $\sigma_{m}^{\prime \prime }\equiv \sum_{j=1}^{3}v_{jm}\sigma_{j}$. It follows that

$$\begin{array}{ccc} \sum_{j,k=1}^{3}T_{jk}\sigma_{j}⊗\sigma_{k} & = & \sum_{m,j,k=1}^{3}s_{jm}d_{m}v_{km}\sigma_{j}⊗\sigma_{k}\\ & = & \sum_{m=1}^{3}d_{m}\sigma_{m}^{\prime }⊗\sigma_{m}^{\prime \prime }. \end{array}$$

Let $\vec{r^{\prime }}\equiv S^{-1}\vec{r}$. It follows that $r_{k}=\sum_{j=1}^{3}s_{kj}r_{j}^{\prime }$ and further $\vec{r}\cdot \vec{\sigma }=\sum_{k,j=1}^{3}s_{kj}r_{j}^{\prime }\sigma_{k}=\sum_{j=1}^{3}r_{j}^{\prime }\sigma_{j}^{\prime }\equiv \vec{r^{\prime }}\cdot \vec{\sigma^{\prime }}$. Similarly, $\vec{s}\cdot \vec{\sigma }=\vec{s^{\prime }}\cdot \vec{\sigma^{\prime \prime }}$ where $\vec{s^{\prime }}\equiv V^{-1}\vec{s}$. Now, we have derived the geometric representation with diagonal correlation matrix in Equation (6). It is trivial to have $tr\left(\sigma_{m}^{\prime }\sigma_{n}^{\prime †}\right)=\sum_{j,k=1}^{3}s_{jm}s_{kn}tr\left(\sigma_{j}\sigma_{k}^{†}\right)=2\sum_{j=1}^{3}s_{jm}s_{jn}=2{\left(S^{T}S\right)}_{mn}=2\delta_{mn}$, which means $\sigma_{1}^{\prime }$, $\sigma_{2}^{\prime }$ and $\sigma_{3}^{\prime }$ are orthogonal to each other. Moreover, it is obviously that $tr(\sigma_{j}^{\prime })=0$. Thus, the operators ${\left\{\sigma_{j}^{\prime }\right\}}_{j=1}^{3}$ plus identity operator form a basis for the operator space of single qubit system. The similar conclusion applies for the operators ${\left\{\sigma_{j}^{\prime \prime }\right\}}_{j=1}^{3}$. We have proved the lemma. □

There are only nine real parameters in the geometric presentation in Equation (6) instead of 15 in Equation (2). Note that any measurement observable on a qubit system can be represented in the new basis as $\vec{a}\cdot \vec{\sigma^{\prime }}$. Therefore, presentation in rotated basis can dramatically simplify analysis procedure in several applications.

### 3.2. Geometric Transformations of Quantum States by Local Unitary Operations

Firstly, we consider the general state of a qubit system which can be completely described by a vector $\vec{r}\in R^{3}$ with $∥\vec{r}∥\leq 1$ as follows,

(7) $$\rho =\frac{1}{2}\left(I+\vec{r}\cdot \vec{\sigma }\right).$$

Suppose unitary operator *U* transforms the state ρ into $\rho^{\prime }$ which is described by a vector $\vec{r^{\prime }}$. Then, it follows that $\vec{r^{\prime }}\cdot \vec{\sigma }=U\vec{r}\cdot \vec{\sigma }U^{†}$ and further $r_{k}^{\prime }=\frac{1}{2}tr\left(\sigma_{k}\vec{r^{\prime }}\cdot \vec{\sigma }\right)=\frac{1}{2}\sum_{j=1}^{3}r_{j}tr\left(\sigma_{k}U\sigma_{j}U^{†}\right)$. Let *V* be a 3×3 matrix with elements defined as follows,

(8) $$V_{kj}\equiv \frac{1}{2}tr\left(\sigma_{k}U\sigma_{j}U^{†}\right).$$

Then, the unitary evolution of the quantum system can be geometrically presented as follows,

(9) $$\vec{r^{\prime }}=V\vec{r}.$$

Note that *V* is a real matrix as $V_{kj}^{*}=\frac{1}{2}tr\left({\left(\sigma_{k}U\sigma_{j}U^{†}\right)}^{†}\right)=V_{kj}$. We call *V* the geometric transformation matrix corresponding to unitary operator *U*.

Now, we consider the local unitary transformation on general two-qubit state. Suppose $U_{A}$ and $U_{B}$ are arbitrary unitary operators on single qubit system. We apply $U_{A}$ and $U_{B}$ on the subsystems of the joint system, which is initialized in the state $\rho_{AB}$ presented in Equation (2), respectively. Let ${\vec{r}}^{\prime }$, ${\vec{s}}^{\prime }$ and $T^{\prime }$ be the geometric parameters of the new derived state $\rho_{AB}^{\prime }\equiv \left(U_{A}⊗U_{B}\right)\rho_{AB}{\left(U_{A}⊗U_{B}\right)}^{†}$. The geometric parameters can be easily obtained by the relation $r_{k}^{\prime }=tr\left(\rho_{AB}^{\prime }\sigma_{k}⊗I\right)$, $s_{k}^{\prime }=tr\left(\rho_{AB}^{\prime }I⊗\sigma_{k}\right)$ and $T_{jk}^{\prime }=tr\left(\rho_{AB}^{\prime }\sigma_{j}⊗\sigma_{k}\right)$. It can be concluded that geometric parameters of the new state are as follows,

(10) $$\begin{array}{c} \left\{\begin{array}{cc} {\vec{r}}^{\prime }= & V_{A}\vec{r},\\ {\vec{s}}^{\prime }= & V_{B}\vec{s},\\ T^{\prime }= & V_{A}TV_{B}^{T}, \end{array}\right. \end{array}$$

where $V_{A}$ and $V_{B}$ are geometric transformation matrices of $U_{A}$ and $U_{B}$ defined as in Equation (8), respectively.

### 3.3. Simplification of Geometric Representation by Local Unitary Operation

In the following, we investigate the correspondence between unitary operation and the geometric transformation matrix. Namely, we find what type of 3×3 matrix that can correspond to a unitary matrix with respect to Equation (8). Finally, we simplify the geometric representation of a general two-qubit state.

Note that $∥\vec{r^{\prime }}{∥}^{2}=\frac{1}{2}tr\left({\left(\vec{r^{\prime }}\cdot \vec{\sigma }\right)}^{2}\right)=\frac{1}{2}tr\left({\left(U\vec{r}\cdot \vec{\sigma }U^{†}\right)}^{2}\right)=\frac{1}{2}tr\left({\left(\vec{r}\cdot \vec{\sigma }\right)}^{2}\right)={∥\vec{r}∥}^{2}$ for any vector $\vec{r}\in R^{3}$. Thus, the geometric transformation matrix *V* must be an orthogonal matrix. However, the converse is not true. For example, there is no unitary matrix on $H^{2}$ corresponding to the orthogonal matrix diag(1,1,−1). Moreover, there are only three diagonal geometric transformation matrices as follows,

(11) $$\begin{array}{c} \left\{\begin{array}{cc} V_{1}= & diag(1,-1,-1),\\ V_{2}= & diag(-1,1,-1),\\ V_{3}= & diag(-1,-1,1), \end{array}\right. \end{array}$$

which correspond to Pauli matrices $\sigma_{1},\sigma_{2},\sigma_{3}$, respectively.

We already know that the unitary operation *U* and the corresponding geometric transformation matrix *V* are related by $U\vec{r}\cdot \vec{\sigma }U^{†}=\left(V\vec{r}\right)\cdot \vec{\sigma }$ for any vector $\vec{r}\in R^{3}$, which can be equivalently written as follows,

(12) $$U\sigma_{k}U^{†}=\sum_{j=1}^{3}V_{jk}\sigma_{j}.$$

Recall that any unitary operator *U* on a qubit system can be written as $U=\cos\theta I-i\sin\theta \vec{\mu }\cdot \vec{\sigma }$ up to some ignorable global phase where $\vec{\mu }\in R^{3}$ is a unit vector and θ∈[0,π). Following Equation (12), we have

(13) $$\begin{array}{cc} & tr\left(U\sigma_{k}\right)=tr\left(\sum_{j=1}^{3}V_{jk}\sigma_{j}U\right) \end{array}$$

(14) $$\begin{array}{cc} \Rightarrow & -i2\sin\theta \mu_{k}=\sum_{j=1}^{3}V_{jk}\left(-i2\sin\theta \mu_{j}\right) \end{array}$$

(15) $$\begin{array}{cc} \Rightarrow & \mu_{k}=\sum_{j=1}^{3}V_{jk}\mu_{j}={\left(V^{T}\vec{\mu }\right)}_{k} \end{array}$$

(16) $$\begin{array}{cc} \Rightarrow & \vec{\mu }=V^{T}\vec{\mu } \end{array}$$

(17) $$\begin{array}{cc} \Rightarrow & \vec{\mu }=V\vec{\mu }. \end{array}$$

Note that the last equation is immediately following from the fact that *V* is an orthogonal matrix. When sinθ=0, *U* is the identity operator on $H^{2}$ and the corresponding geometric transformation matrix is the identity operator on $R^{3}$. Therefore, the rotation axis of any unitary operator on $H^{2}$ is the eigenvector of the corresponding geometric transformation matrix and the corresponding eigenvalue is 1.

According to Equation (8), we get the diagonal elements of matrix *V* as follows,

$$\begin{array}{ccc} V_{kk} & = & \frac{1}{2}tr\left(\sigma_{k}U\sigma_{k}U^{†}\right)\\ & = & \frac{1}{2}\left(2\cos^{2}\theta +\sin^{2}\theta tr\left(\sigma_{k}\left(\vec{\mu }\cdot \vec{\sigma }\right)\sigma_{k}\left(\vec{\mu }\cdot \vec{\sigma }\right)\right)\right)\\ & = & \cos^{2}\theta +\sin^{2}\theta \left(2\mu_{k}^{2}-1\right)\\ & = & 1+2\sin^{2}\theta \left(\mu_{k}^{2}-1\right). \end{array}$$

Then, it is trivial to obtain that the trace of the matrix *V* is tr(V)=1+2cos2θ. Recall that the eigenvalues of any orthogonal matrix are ±1 and pairs of $\left(e^{i\alpha },e^{-i\alpha }\right)$. The eigenvalues of the geometric transformation matrix *V* must be 1, $e^{i\alpha }$ and $e^{-i\alpha }$ for some parameter α∈[0,2π). Further, the trace of *V* should be tr(V)=1+2cosα. Therefore, the rotation angle of unitary operator *U* that corresponds to geometric transformation matrix *V* is $\frac{1}{2}\arccos\frac{tr(V)-1}{2}$. We conclude the above discussion as the following theorem.

**Theorem** **1.** *Any matrix V is the geometric transformation matrix of a unitary operator on $H^{2}$ if and only if V is a 3×3 real orthogonal matrix with eigenvalues 1, $e^{2i\theta }$ and $e^{-2i\theta }$. The unitary operator corresponding to V is $U=\cos\theta I-i\sin\theta \vec{\mu }\cdot \vec{\sigma }$ where $\vec{\mu }$ is the eigenvector of V corresponding to eigenvalue 1.*

The Equation (12) holds for any k∈{1,2,3}. Suppose (k,j,t) is a permutation of (1,2,3) such that $\sigma_{j}\sigma_{t}=i\sigma_{k}$. Namely, (k,j,t)∈{(1,2,3),(2,3,1),(3,1,2)}. Then, we can get $tr\left(U\right)=tr(\left(\sum_{j=1}^{3}V_{jk}\sigma_{j}\right)U\sigma_{k})$, which is finally reduced to the relation $2\cos\theta =2\cos\theta V_{kk}+2\sin\theta \left(V_{jk}\mu_{t}-V_{tk}\mu_{j}\right)$. Thus, we can get the rotation angle θ via the equation as follows,

(18) $$\cot\theta =\frac{V_{jk}\mu_{t}-V_{tk}\mu_{j}}{1-V_{kk}},$$

where the diagonal element $V_{kk}\neq 1$.

Now we investigate the simplification of the general two-qubit state $\rho_{AB}$ presented in Equation (2). Suppose the singular value decomposition of the correlation matrix is $T=S\Sigma D^{T}$ where Σ is a real diagonal matrix, *S* and *D* are 3×3 real orthogonal matrices. Note that $S^{T}$ is also an orthogonal matrix and has at least one eigenvalue being +1 or −1. Firstly, we consider the case that 1 is an eigenvalue of $S^{T}$. According Theorem 1, $S^{T}$ is the geometric transformation matrix of the unitary operator $U_{A}=\cos\theta I-i\sin\theta \vec{\mu }\cdot \vec{\sigma }$ where $\theta =\frac{1}{2}\arccos\frac{tr(S)-1}{2}$ and $\vec{\mu }$ is the eigenvector of $S^{T}$ corresponding to the eigenvalue 1. If $S^{T}$ has an eigenvalue -1, then the orthogonal matrix −S has an eigenvalue 1 and we can view the singular value decomposition of *T* as $T=\left(-S\right)\left(-\Sigma \right)D^{T}$ where $-S^{T}$ is a geometric transformation matrix. Similarly, we can find a unitary operator $U_{B}$ that corresponding to the geometric transformation matrix *D* or −D. Applying $U_{A}$ and $U_{B}$ on subsystems *A* and *B*, respectively, the correlation matrix of the joint system will become the diagonal matrix Σ. Therefore, we can always find local unitary operations to transform any two-qubit state into the one with diagonal correlation matrix. By applying geometric transformation matrices of Puali matrices in Equation (11), there are at least two positive elements in the diagonal correlation matrix.

We have proved the following lemma.

**Lemma** **2.** *By applying local unitary operations, any two-qubit state can be transformed into the one with diagonal correlation matrix as follows,*

(19) $$\begin{array}{cc} \rho_{AB}=\frac{1}{4}(I⊗I+\vec{r}\cdot \vec{\sigma }⊗I+I⊗\vec{s}\cdot \vec{\sigma } & +\sum_{k=1}^{3}t_{k}\sigma_{k}^{⊗2}), \end{array}$$

*where at least two of the diagonal elements $\left\{t_{1},t_{2},t_{3}\right\}$ are non-negative.*

## 4. Geometric Bounds for Concurrence

Although we can analytically measure quantum entanglement by concurrence, it is difficult to understand entanglement in perspective of geometric approach. In this section, we find both upper bound and lower bound for concurrence in geometric parameters.

**Theorem** **2.** *Suppose ρ is a general two-qubit state with geometric parameters $\left(T,\vec{r},\vec{s}\right)$. Then, the concurrence of ρ is bounded as follows,*

(20) $$\frac{1}{2}{(∥T∥}_{KF}-1)\leq C\left(\rho \right)\leq \sqrt{1-\max\{∥\vec{r}{∥}^{2},∥\vec{s}{∥}^{2}\}},$$

*where ${∥T∥}_{KF}\equiv tr\left(\sqrt{T^{T}T}\right)$ is the Ky Fan matrix norm of T.*

**Proof.**  According to the discussion in Section 2.2, we can assume that $\rho =\sum_{k}p_{k}|\psi_{k}\rangle \langle \psi_{k}|$ is a decomposition such that

(21) $$C\left(\psi_{k}\right)=C\left(\rho \right)$$

for all pure state $|\psi_{k}\rangle$. Further suppose the geometric parameters for the pure state $|\psi_{k}\rangle$ are $(T_{k},\vec{r_{k}},\vec{s_{k}}$).

Note that any pure state of a two-qubit system can be transformed into $|\Phi_{\theta }\rangle =\cos\theta |00\rangle +\sin\theta |11\rangle$ for some $\theta \in [-\frac{\pi }{2},\frac{\pi }{2}]$ by local unitary operations. It is trivial to find that the geometric parameters of $|\Phi_{\theta }\rangle$ are $\vec{r_{\theta }}=\vec{s_{\theta }}=\left(0,0,\cos2\theta \right)$ and $T_{\theta }=diag(\sin2\theta$, −sin2θ,1). The concurrence of $|\Phi_{\theta }\rangle$ is $C(\Phi_{\theta })=|\sin2\theta |$. The correlation matrix is related to concurrence by the equation

(22) $$C\left(\Phi_{\theta }\right)=\frac{1}{2}(∥T_{\theta }{∥}_{KF}-1)$$

and the local parameter is related to the concurrence by the equations

(23) $$C\left(\Phi_{\theta }\right)=\sqrt{1-∥\vec{r_{\theta }}{∥}^{2}}=\sqrt{1-∥\vec{s_{\theta }}{∥}^{2}}.$$

Combing Equations (21) and (22), it follows that

(24) $$\begin{array}{ccc} C(\rho ) & = & \sum_{k}p_{k}C\left(\psi_{k}\right) \end{array}$$

(25) $$\begin{array}{ccc} & = & \frac{1}{2}(\sum_{k}p_{k}∥T_{k}{∥}_{KF}-1) \end{array}$$

(26) $$\begin{array}{ccc} & \geq & \frac{1}{2}(∥\sum_{k}p_{k}T_{k}{∥}_{KF}-1) \end{array}$$

(27) $$\begin{array}{ccc} & = & \frac{1}{2}{(∥T∥}_{KF}-1). \end{array}$$

where the inequality (26) follows from the subadditivity of norm. We have proved the lower bound of concurrence.

The Equations (21) and (23) implies that $∥\vec{r_{k}}∥=∥\vec{r_{j}}∥$ for all component states $|\psi_{k}\rangle$ and $|\psi_{j}\rangle$. Then, it follows that

(28) $$\begin{array}{ccc} ∥\vec{r}∥ & = & ∥\sum_{j}p_{j}\vec{r_{j}}∥ \end{array}$$

(29) $$\begin{array}{ccc} & \leq & \sum_{j}p_{j}∥\vec{r_{j}}∥ \end{array}$$

(30) $$\begin{array}{ccc} & = & ∥\vec{r_{k}}∥ \end{array}$$

for pure state $|\psi_{k}\rangle$. Further, we can get

(31) $$\begin{array}{ccc} C(\rho ) & = & \sum_{k}p_{k}\sqrt{1-∥\vec{r_{k}}{∥}^{2}} \end{array}$$

(32) $$\begin{array}{ccc} & \leq & \sum_{k}p_{k}\sqrt{1-∥\vec{r}{∥}^{2}} \end{array}$$

(33) $$\begin{array}{ccc} & = & \sqrt{1-∥\vec{r}{∥}^{2}}. \end{array}$$

Similarly, we can get $C\left(\rho \right)\leq \sqrt{1-∥\vec{s}{∥}^{2}}$. Therefore, we have proved the following upper bound

(34) $$C\left(\rho \right)\leq \sqrt{1-\max\{∥\vec{r}{∥}^{2},∥\vec{s}{∥}^{2}\}}.$$

Clearly, the upper bound and lower bound can be achieved when ρ is a pure state. □

Via numerical experiment, we find another upper bound as follows,

(35) $$C\left(\rho \right)\leq \frac{1}{2}\sqrt{1+{∥T∥}_{F}^{2}-∥\vec{r}{∥}^{2}-{∥\vec{s}∥}^{2}},$$

where we denote ${∥T∥}_{F}\equiv \sqrt{tr(T^{T}T)}=\sqrt{\sum_{j,k}T_{jk}^{2}}$. Note that this is a tighter upper bound than the one $C\left(\rho \right)\leq \sqrt{1-\frac{1}{2}(∥\vec{r}{∥}^{2}+∥\vec{s}{∥}^{2})}$, which can be derived by the constraint of geometric parameters in Equation (36).

## 5. The Local Parameters and Separability

In this section, we investigate how the local parameters affect the separability of a two-qubit state. Because of the equivalence of local unitary operations in the separability problem, our analysis only focus on the states with diagonal correlation matrix in Equation (19).

Note that we denote the vector $\vec{t}=\left(t_{1},t_{2},t_{3}\right)$ instead of a diagonal correlation matrix *T*. Let the local parameters be represented by $\vec{r}=\left(r_{1},r_{2},r_{3}\right)$ and $\vec{s}=\left(s_{1},s_{2},s_{3}\right)$. We are unable to depict the analytical conditions for the general parameters $\vec{r},\vec{s}$ and $\vec{t}$ such that $\rho_{AB}$ is a density operator. However, a necessary condition is $tr(\rho_{AB}^{2})\leq 1$, which can be equivalently written as

(36) $$∥\vec{r}{∥}^{2}+∥\vec{s}{∥}^{2}+{∥\vec{t}∥}^{2}\leq 3.$$

Meanwhile, the local parameters must satisfy $∥\vec{r}∥\leq 1$ and $∥\vec{s}∥\leq 1$. Further, we suppose $\rho_{AB}=\sum_{k=1}^{4}\lambda_{k}|\psi_{k}\rangle \langle \psi_{k}|$ is the spectral decomposition of the density operator $\rho_{AB}$.

### 5.1. Permutation of Pauli Matrices by Local Unitary

We have showed that any two-qubit state can be local unitary equivalently transformed to a state with diagonal correlation matrix, say $T=diag(t_{1},t_{2},t_{3})$. To investigate the role that each parameters $t_{k}$ played in the separability of the state, we want to ask the question whether there is any local unitary that can permutate Pauli matrices. We assume that there is a unitary operator $U_{jkt}$ on a single qubit system such that

(37) $$\begin{array}{c} \left\{\begin{array}{cc} U_{jkt}\sigma_{1}U_{jkt}^{†} & =\sigma_{j},\\ U_{jkt}\sigma_{2}U_{jkt}^{†} & =\sigma_{k},\\ U_{jkt}\sigma_{3}U_{jkt}^{†} & =\sigma_{t}, \end{array}\right. \end{array}$$

where (j,k,t) is a permutation of (1,2,3). Note that the permutation (j,k,t) has six possible choices, namely {(1,2,3),(1,3,2),(2,1,3),(2,3,1),(3,1,2),(3,2,1)}. It is trivial to see that $U_{123}=I$ as it keeps every Pauli operator invariant. Suppose that the geometric rotation matrix corresponding to $U_{jkt}$ is $V_{jkt}$. According to Equation (37), the ${\left(j,1\right)}^{th}$ element of matrix $V_{jkt}$ is ${\left(V_{jkt}\right)}_{j1}=\frac{1}{2}tr\left(\sigma_{j}U_{jkt}\sigma_{1}U_{jkt}^{†}\right)=\frac{1}{2}tr\left(\sigma_{j}\sigma_{j}\right)=1$. Similarly, we get ${\left(V_{jkt}\right)}_{k2}={\left(V_{jkt}\right)}_{t3}=1$ and the other elements are 0. Note that the matrix $V_{jkt}$ is a 3×3 orthonormal matrix. Thus, there must be an unitary matrix $U_{jkt}$ corresponding to $V_{jkt}$. The nontrivial permutations can be classified into two classes. The first class are full permutations which include (2,3,1) and (3,1,2). The corresponding unitary operators are as follows,

(38) $$\begin{array}{c} U_{231}=\frac{1}{2}I-i\frac{1}{2}\left(X+Y+Z\right), \end{array}$$

(39) $$\begin{array}{c} U_{312}=\frac{1}{2}I+i\frac{1}{2}\left(X+Y+Z\right). \end{array}$$

The second class partially permutates the Pauli matrices where only one Pauli matrix is kept invariant and the other two are exchanged. Suppose unitary $U_{k}$ corresponds to the permutation that keeps $\sigma_{k}$ invariant. Then, the unitary operator $U_{k}$ is of the form as follows,

(40) $$U_{k}=\frac{1}{\sqrt{2}}\left(I-i\sigma_{k}\right).$$

To be consistent with the aforementioned definitions, we have $U_{132}=U_{1}$, $U_{321}=U_{2}$ and $U_{213}=U_{3}$. Note that if (k,j,t) is in the order such that $\sigma_{k}\sigma_{j}=i\sigma_{t}$, $U_{k}$ transforms $\sigma_{t}$ into $\sigma_{j}$ up to a phase −1. Otherwise, it takes $\sigma_{j}$ into $\sigma_{t}$ up to a phase −1.

### 5.2. Two Classes of Separable States

In this subsection, we show two classes of separable states based on special geometric parameters.

First, we look at the two-qubit state with vanished correlation parameters $\vec{t}=\left(0,0,0\right)$ and general local parameters $\vec{r}$ and $\vec{s}$. In this case, the eigenvalues of the operator $\rho_{AB}$ are $\frac{1}{4}(1\pm |∥\vec{r}∥\pm ∥\vec{s}∥|)$. Thus, $\rho_{AB}$ is positive if and only if $∥\vec{r}∥+∥\vec{s}∥|\leq 1$. Further, we find that the eigenvalues of the operator $\rho_{AB}$ is exactly the same as that of its partial transposition $\rho_{AB}^{PT}$. Thus, the positivity of $\rho_{AB}^{PT}$ is equivalent to the condition that the operator $\rho_{AB}$ is a density operator. Therefore, the two-qubit state with vanished correlation matrix is separable. We have proved the following lemma.

**Lemma** **3.** *The operator $\rho_{AB}=\frac{1}{4}\left(I⊗I+\vec{r}\cdot \vec{\sigma }⊗I+I⊗\vec{s}\cdot \vec{\sigma }\right)$ is a density operator if and only if $∥\vec{r}∥+∥\vec{s}∥\leq 1$. Any density operator of this form is separable.*

As a trivial application of partial transposition criterion, Theorem 3 reveals a class of separable two-qubit states with respect to local parameters.

**Theorem** **3.** *The density operator in Equation (19) is separable if $t_{k}=0$ and $r_{k}s_{k}=0$ for any k=1,2,3.*

**Proof.**  According to the PPT criterion [20] and its extended research [21], the density operator $\rho_{AB}$ is separable if and only if the partial transposition $\rho_{AB}^{PT}$ is positive. As the density operator $\rho_{AB}$ is positive, the operator $\rho_{AB}^{PT}$ is positive if $\rho_{AB}^{PT}=\rho_{AB}$, which is equivalent to $t_{2}=r_{2}=0$. Similarly, the operator $\rho_{AB}$ is separable if $\rho_{BA}^{PT}=\rho_{BA}$, which is equivalent to $t_{2}=s_{2}=0$. Thus, the state $\rho_{AB}$ is separable if $t_{2}=0$ and $r_{2}s_{2}=0$.

Applying local unitary operators $U_{213}⊗U_{213}$ defined in Equation (40) on state $\rho_{AB}$, the geometric parameters of the derived state $\rho_{AB}^{\prime }=\left(U_{213}⊗U_{213}\right)\rho_{AB}{\left(U_{213}⊗U_{213}\right)}^{†}$ are $\vec{t^{\prime }}=\left(t_{2},t_{1},t_{3}\right)$, $\vec{r^{\prime }}=\left(r_{2},r_{1},r_{3}\right)$ and $\vec{s^{\prime }}=\left(s_{2},s_{1},s_{3}\right)$. The condition $t_{2}=0$ and $r_{2}s_{2}=0$ is equivalent to $t_{1}^{\prime }=0$ and $r_{1}^{\prime }s_{1}^{\prime }=0$. As local unitary operations do not affect separability, the separability of states $\rho_{AB}^{\prime }$ and $\rho_{AB}$ are same. Thus, $t_{1}^{\prime }=0$ and $r_{1}^{\prime }s_{1}^{\prime }=0$ is a sufficient condition that $\rho_{AB}^{\prime }$ is a separable state. As $\rho_{AB}^{\prime }$ is a general state, we can also say that $\rho_{AB}$ is separable if $t_{1}=0$ and $r_{1}s_{1}=0$. Similarly, it is trivial to show that the state $\rho_{AB}$ is separable if $t_{3}=0$ and $r_{3}s_{3}=0$.

Therefore, we can conclude that the density operator $\rho_{AB}$ defined in Equation (19) is separable if $t_{k}=0$ and $r_{k}s_{k}=0$ for any k∈{1,2,3}. □

### 5.3. Local Parameters as an Indispensable Role for Entanglement

In this subsection, we investigate the separability of a two-quibt system that can be affected by local parameters. We consider the class of two-qubit states with geometric parameters $\vec{r}=\left(0,r_{2},0\right)$, $\vec{s}=\left(0,s_{2},0\right)$ and $\vec{t}=\left(t_{1},t_{2},t_{3}\right)$. Because of unitary operators for permutating Pauli matrices discussed in Section 5.1, the above results can be generalized for two-qubit states where non-vanished local parameters are $r_{k}$ and $s_{k}$ for any k=1,2,3.

The eigenvalues of the corresponding density operator $\rho =\frac{1}{4}\left(I⊗I+r_{2}\sigma_{2}⊗I+s_{2}I⊗\sigma_{2}+\sum_{k=1}^{3}t_{k}\sigma_{k}⊗\sigma_{k}\right)$ are as follows,

(41) $$\begin{array}{c} \left\{\begin{array}{ccc} \lambda_{1} & = & \frac{1}{4}\left(1-t_{2}-\sqrt{{\left(r_{2}-s_{2}\right)}^{2}+{\left(t_{1}+t_{3}\right)}^{2}}\right),\\ \lambda_{2} & = & \frac{1}{4}\left(1+t_{2}+\sqrt{{\left(r_{2}+s_{2}\right)}^{2}+{\left(t_{1}-t_{3}\right)}^{2}}\right),\\ \lambda_{3} & = & \frac{1}{4}\left(1+t_{2}-\sqrt{{\left(r_{2}+s_{2}\right)}^{2}+{\left(t_{1}-t_{3}\right)}^{2}}\right),\\ \lambda_{4} & = & \frac{1}{4}\left(1-t_{2}+\sqrt{{\left(r_{2}-s_{2}\right)}^{2}+{\left(t_{1}+t_{3}\right)}^{2}}\right). \end{array}\right. \end{array}$$

The positivity of density operator ρ requires $\lambda_{k}\geq 0$ for every k=1,2,3,4, which is equivalently constrained by the inequalities as follows,

(42) $$\begin{array}{cc} {\left(r_{2}-s_{2}\right)}^{2}+{\left(t_{1}+t_{3}\right)}^{2}\leq & {\left(1-t_{2}\right)}^{2},\;\mathrm{and} \end{array}$$

(43) $$\begin{array}{cc} {\left(r_{2}+s_{2}\right)}^{2}+{\left(t_{1}-t_{3}\right)}^{2}\leq & {\left(1+t_{2}\right)}^{2}. \end{array}$$

From above restrictions, it is obvious to get a necessary condition for being a density operator as follows,

(44) $$r_{2}^{2}+s_{2}^{2}\leq 1+t_{2}^{2}-t_{1}^{2}-t_{3}^{2}.$$

Suppose ${\left\{\mu_{k}\right\}}_{k=1}^{4}$ are eigenvalues of the operator $\sqrt{\rho \tilde{\rho }}$ where $\tilde{\rho }\equiv \left(Y⊗Y\right)\rho^{*}\left(Y⊗Y\right)$ and $\rho^{*}$ is the complex conjugate of ρ. Let $C\left(\rho \right)\equiv \max_{k}\left(2\mu_{k}-\sum_{j=1}^{4}\mu_{j}\right)$. Then, the concurrence of ρ is max{0,C(ρ)} and ρ is entangled if and only if C(ρ)>0 [28]. Via trivial calculation, we get the eigenvalues of $\sqrt{\rho \tilde{\rho }}$ as follows,

(45) $$\begin{array}{c} \left\{\begin{array}{ccc} \mu_{1} & = & \frac{1}{4}\left(\sqrt{{\left(1+t_{2}\right)}^{2}-{\left(r_{2}+s_{2}\right)}^{2}}-\left(t_{1}-t_{3}\right)\right),\\ \mu_{2} & = & \frac{1}{4}\left(\sqrt{{\left(1+t_{2}\right)}^{2}-{\left(r_{2}+s_{2}\right)}^{2}}+\left(t_{1}-t_{3}\right)\right),\\ \mu_{3} & = & \frac{1}{4}\left(\sqrt{{\left(1-t_{2}\right)}^{2}-{\left(r_{2}-s_{2}\right)}^{2}}-\left(t_{1}+t_{3}\right)\right),\\ \mu_{4} & = & \frac{1}{4}\left(\sqrt{{\left(1-t_{2}\right)}^{2}-{\left(r_{2}-s_{2}\right)}^{2}}+\left(t_{1}+t_{3}\right)\right). \end{array}\right. \end{array}$$

It is trivial to see that the state is separable if and only if $2\mu_{k}-\sum_{j=1}^{4}\mu_{j}\leq 0$ for all k=1,2,3,4, which is equivalent to the conditions as follows,

(46) $$\begin{array}{cc} {\left(r_{2}-s_{2}\right)}^{2}+{\left(t_{1}-t_{3}\right)}^{2}\leq & {\left(1-t_{2}\right)}^{2},\;\mathrm{and} \end{array}$$

(47) $$\begin{array}{cc} {\left(r_{2}+s_{2}\right)}^{2}+{\left(t_{1}+t_{3}\right)}^{2}\leq & {\left(1+t_{2}\right)}^{2}. \end{array}$$

Obviously, the state is entangled if $|t_{1}-t_{3}|>|1-t_{2}|$ or $|t_{1}+t_{3}|>|1+t_{2}|$, which is equivalent to the entanglement condition showed by Horodeckis for the states with vanished local variables [15].

To investigate the role of local parameters for entanglement, we suppose the density operator $\rho (r_{2},s_{2})$ is a function of local parameters $r_{2}$ and $s_{2}$ with $t_{1}$, $t_{2}$ and $t_{3}$ being fixed correlation parameters. Then, the state is entangled if $C(\rho \left(r_{2},s_{2}\right))>0$, namely either Equation (46) or Equation (47) is violated. In the case Equation (46) is violated, the concurrence is $C\left(\rho \left(r_{2},s_{2}\right)\right)=\frac{1}{2}\left(\right|t_{1}-t_{3}\left|-\sqrt{{\left(1-t_{2}\right)}^{2}-{\left(r_{2}-s_{2}\right)}^{2}}\right)$, which is an increasing function in $|r_{2}-s_{2}|$. The positivity conditions for density operators can be equivalently written as follows,

(48) $$\begin{array}{cc} {\left(r_{2}-s_{2}\right)}^{2}\leq & {\left(1-t_{2}\right)}^{2}-{\left(t_{1}+t_{3}\right)}^{2}\equiv \epsilon_{0},\;\mathrm{and} \end{array}$$

(49) $$\begin{array}{cc} {\left(r_{2}-s_{2}\right)}^{2}\leq & {\left(1+t_{2}\right)}^{2}-{\left(t_{1}-t_{3}\right)}^{2}-4r_{2}s_{2}\equiv \epsilon_{1}. \end{array}$$

Suppose the equality in Equation (48) holds. We can get $r_{2}^{2}+s_{2}^{2}-\left(1+t_{2}^{2}-t_{1}^{2}-t_{3}^{2}\right)=2\left(r_{2}s_{2}-t_{2}-t_{1}t_{3}\right)\leq 0$ which is immediate following from Equation (44). Then, $\epsilon_{1}-\epsilon_{0}=-4\left(r_{2}s_{2}-t_{2}-t_{1}t_{3}\right)\geq 0$, which indicates that the positivity condition of density operator in Equation (49) naturally holds when $|r_{2}-s_{2}|$ takes the upper bound in Equation (48). Therefore, the concurrence takes the maximum value when $|r_{2}-s_{2}|=\sqrt{{\left(1-t_{2}\right)}^{2}-{\left(t_{1}+t_{3}\right)}^{2}}$, namely $C\left(\rho \left(r_{2},s_{2}\right)\right)\leq \frac{1}{2}\left(\right|t_{1}-t_{3}\left|-\right|t_{1}+t_{3}\left|\right)$. Combining Equations (42) and (43) and the violation of Equation (46), it is sufficient to have $t_{1}t_{3}<0$ and $r_{2}s_{2}<t_{2}$. A trivial calculation shows that $C\left(\rho \left(r_{2},s_{2}\right)\right)\leq \min\{|t_{1}\left|,\right|t_{2}\left|\right\}$.

The similar result can be obtained for the case that the second inequality is violated. Suppose the state is entangled because of the violation of Equation (47). The concurrence of the system is $C\left(\rho \left(r_{2},s_{2}\right)\right)=\frac{1}{2}\left(\right|t_{1}+t_{3}\left|-\sqrt{{\left(1+t_{2}\right)}^{2}-{\left(r_{2}+s_{2}\right)}^{2}}\right)$, which is an increasing in $|r_{2}+s_{2}|$. The maximum of $C(\rho \left(r_{2},s_{2}\right))$ is also $\min\{|t_{1}|,|t_{2}|\}$, which can be obtained when $|r_{2}+s_{2}|=\sqrt{{\left(1+t_{2}\right)}^{2}-{\left(t_{1}-t_{3}\right)}^{2}}$. It is also trivial to find that $t_{1}t_{3}>0$ and $r_{2}s_{2}>t_{2}$.

We have proved the following theorem.

**Theorem** **4.** *Suppose $\rho \left(r_{2},s_{2}\right)=\frac{1}{4}\left(I⊗I+r_{2}\sigma_{2}⊗I+s_{2}I⊗\sigma_{2}+\sum_{k=1}^{3}t_{k}\sigma_{k}⊗\sigma_{k}\right)$ is a density operator of a two-qubit system. The system is entangled if and only if either inequality in Equation (46) or Equation (47) is violated. The corresponding concurrence is $C\left(\rho \left(r_{2},s_{2}\right)\right)=\max\{\frac{1}{2}\left(\right|t_{1}\pm t_{3}|-\sqrt{{\left(1\pm t_{2}\right)}^{2}-{\left(r_{2}\pm s_{2}\right)}^{2}})\}$ which is an increasing function in $|r_{2}\pm s_{2}|$. The upper bound on concurrence is as follows,*

(50) $$C\left(\rho \left(r_{2},s_{2}\right)\right)\leq \min\{|t_{1}\left|,\right|t_{2}\left|\right\},$$

*where the equality holds when $|r_{2}\pm s_{2}|=\sqrt{{\left(1\pm t_{2}\right)}^{2}-{\left(t_{1}\mp t_{3}\right)}^{2}}$. $t_{1}t_{3}\left(t_{2}-r_{2}s_{2}\right)<0$ is a necessary condition that $\rho (r_{2},s_{2})$ is entangled.*

To quantitatively analyze the separability, we have the following theorem.

**Theorem** **5.** *Consider two-qubit state with nonvanished local parameters $r_{2}$, $s_{2}$ and correlation parameters $t_{1},t_{2},t_{3}$. The state is separable for any local parameters if and only if $t_{1}t_{3}=0$. We can always find a local parameter $r_{2}$ for any valid local parameter $s_{2}$ such that the state is entangled if and only if the correlation parameters $t_{1}t_{2}t_{3}<0$.*

**Figure 1:**
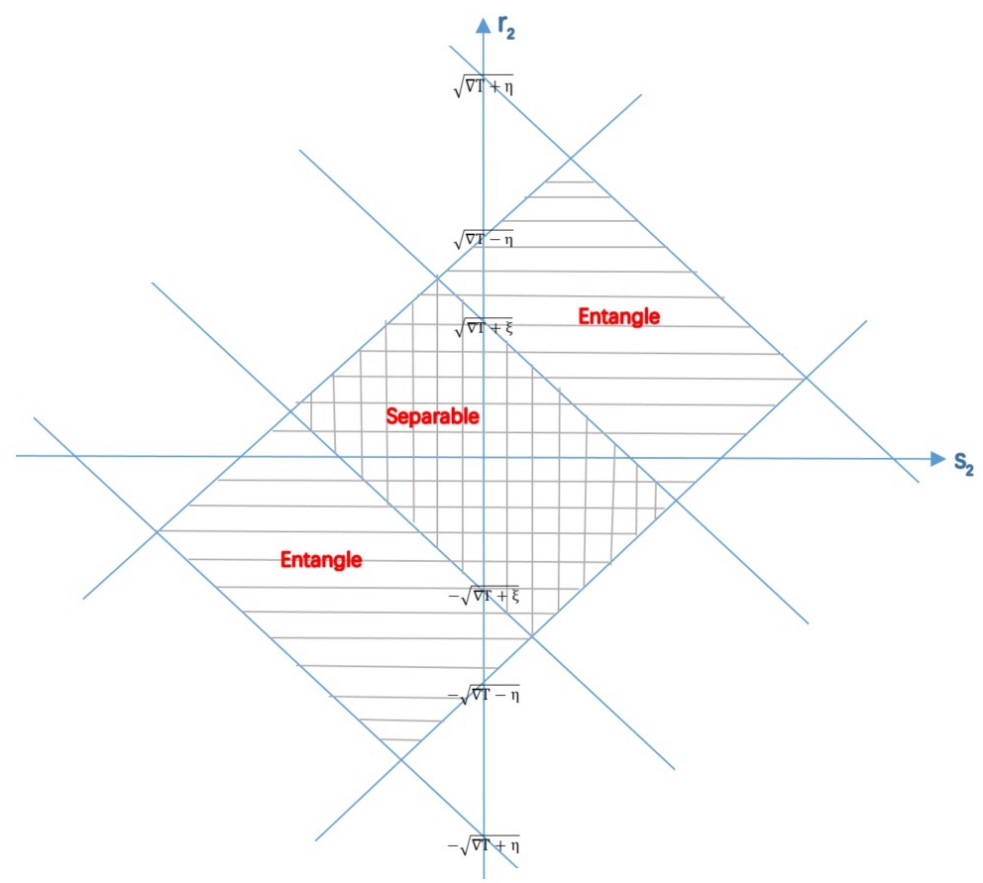
Separability of a two-qubit state affected by local parameters. Here we define $\Delta T\equiv 1-t_{1}^{2}+t_{2}^{2}-t_{3}^{2}$, $\eta \equiv 2(t_{2}+t_{1}t_{3})$ and $\xi \equiv 2(t_{2}-t_{1}t_{3})$. With out loss of generality, we suppose $t_{2}+t_{1}t_{3}\geq 0$.

**Proof.**  Let $\Delta T\equiv 1-t_{1}^{2}+t_{2}^{2}-t_{3}^{2}$, $\eta \equiv 2(t_{2}+t_{1}t_{3})$ and $\xi \equiv 2(t_{2}-t_{1}t_{3})$. We suppose η≥0 with out loss of generality. Then, the operator is a density operator if and only if $|r_{2}\pm s_{2}|\leq \sqrt{\Delta T\pm \eta }$ while it is separable if and only if $|r_{2}\pm s_{2}|\leq \sqrt{\Delta T\pm \xi }$, which can be depicted in Figure 1.

The state is always separable for any local parameters if and only if the area of Equations (46) and (47) include the area of valid state, namely ξ≥η and −ξ≥−η, which is equivalent to $t_{1}t_{3}=0$. This result is consistent with Theorem 3.

The state is possible to be entangled for any local parameter $s_{2}$ if and only if −ξ<−η, which is equivalent to $t_{1}t_{3}<0$. According to the assumption $t_{2}+t_{1}t_{3}\geq 0$, we further have $t_{2}>0$. Thus, it is necessary to have $t_{1}t_{2}t_{3}<0$, which also applies for all other possible cases. Therefore, we conclude that we can always find a local parameter $r_{2}$ for any valid local parameter $s_{2}$ such that the state is entangled if and only if $t_{1}t_{2}t_{3}<0$.

The same result can be obtained in the case η>0. □

**Figure 2:**
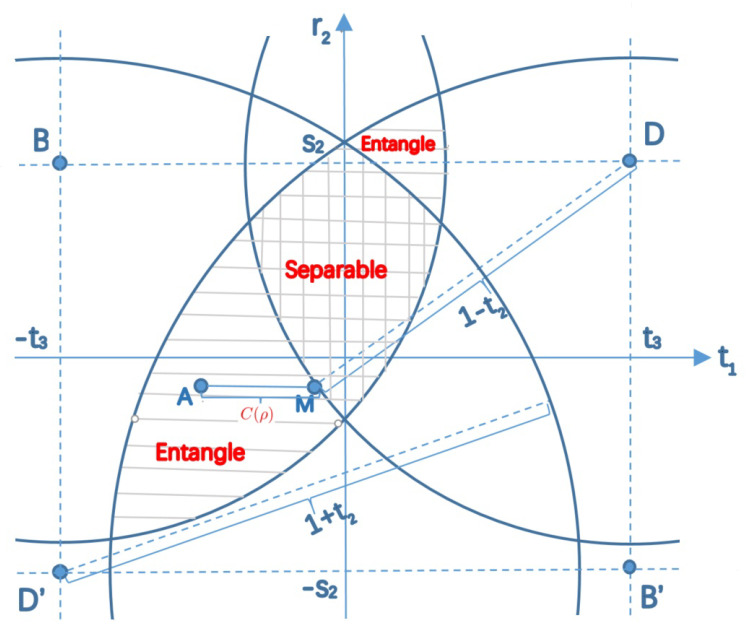
The separability of a two-qubit states with geometric parameters being $\vec{r}=\left(0,r_{2},0\right)$, $\vec{s}=\left(0,s_{2},0\right)$ and $\vec{t}=\left(t_{1},t_{2},t_{3}\right)$. The point $A(t_{1},r_{2})$ is a free variable. The operator $\rho_{AB}$ is a density operator iff *A* is in the area with horizontal lines. The state is separable iff *A* is in the area with vertical lines. The state is entangled iff *A* is in the area only with horizontal lines and the corresponding concurrence is $C(\rho_{AB})=|AM|$ or $C(\rho_{AB})=|AN|$ depending the location of *A*.

We can also illustrate the separability of the state ρ with respect to free variables $t_{1}$ and $r_{2}$ in a two-dimensional Cartesian coordinate system as showed in Figure 2. According to Lemma 2, we assume $t_{1}$ and $t_{2}$ are non-negative. We also assume $s_{2}>0$ with out loss of generality. Let $A=(t_{1},r_{2})$ be a free point and $B=(-t_{3},s_{2})$, $B^{\prime }=\left(t_{3},-s_{2}\right)$, $D=(t_{3},s_{2})$ and $D^{\prime }=\left(-t_{3},-s_{2}\right)$ be fixed points on the plane.

In the following, we also use circle *B* to denote the circle with the center at point *B* and radius $1-t_{2}$. Similarly, circle *D* is a circle with radius $1-t_{2}$, and circle $B^{\prime }$ and circle $D^{\prime }$ are circles with radius $1+t_{2}$. Then, the operator ρ is a density operator if and only if $\left|AB\right|\leq 1-t_{2}$ and $|AB^{\prime }|\leq 1+t_{2}$, namely the free point *A* is in the intersection of circle *B* and circle $B^{\prime }$ which is depicted with horizontal lines. ρ is separable if and only if $\left|AD\right|\leq 1-t_{2}$ and $|AD^{\prime }|\leq 1+t_{2}$, namely the free point *A* is in the intersection of circle *D* and $D^{\prime }$ which is depicted with vertical lines. Obviously, the state ρ is entangled if and only if the free point *A* is in the area with horizontal lines only.

Now we quantitatively analyze the entanglement of the state ρ. If the free point *A* is in the left below entangled area, the concurrence of ρ is $C\left(\rho \right)=|t_{1}-t_{3}|-\sqrt{{\left(1-t_{2}\right)}^{2}-{\left(r_{2}-s_{2}\right)}^{2}}$.

Suppose the projection of *A* onto the vertical axis is intersected with the circle *D* with radius $1-t_{2}$ at point *M*. Then, the concurrence C(ρ)=|AM|. If *A* is a point in the top right entangled area, the concurrence of the state ρ is $C\left(\rho \right)=|t_{1}+t_{3}|-\sqrt{{\left(1+t_{2}\right)}^{2}-{\left(r_{2}+s_{2}\right)}^{2}}$. Suppose the projection of *A* on to the vertical axis is intersected with the circle $D^{\prime }$ with radius $1+t_{2}$ at point *M*. Then, the concurrence C(ρ)=|AN|. In both cases, the entanglement reach the maximum when point *A* is located at the intersection of circle *B* and circle $B^{\prime }$, namely when the equations in Equations (42) and (43) hold.

We conclude above discussion as the following lemma.

**Lemma** **4.** *Suppose $\rho =\frac{1}{4}\left(I⊗I+r_{2}\sigma_{2}⊗I+s_{2}I⊗\sigma_{2}+\sum_{k=1}^{3}t_{k}\sigma_{k}⊗\sigma_{k}\right)$ is a two-qubit state as geometrically depicted in Figure 2 without loss of generality. The state ρ is entangled if and only if the free point A is only in the circle D or $D^{\prime }$. The concurrence of state ρ is C(ρ)=|AM| where M is the intersection of point A’s projection onto the vertical axis and the other circle which A is not in.*

## 6. Conclusions and Discussion

We have considered the role of local geometric parameters on the entanglement of bipartite quantum system. We found that the local parameters of a two-qubit system have significant impact on its separability. We simplified the analysis by considering an entanglement equivalent form with reduced number of geometric parameters. We conclude our contribution in three-fold: (1) we found that the concurrence of a general two-qubit state is bounded by the norms of local vectors and correlations matrix; (2) we derived a sufficient condition that the state is separable based on the PPT criterion; (3) we found that a quantum state with fixed correlation matrix can be entangled or separable depending on different values of local parameters.

We made the third conclusion by investigating a special state with general correlation matrix and local parameters $r_{2}$ and $s_{2}$. We found that the entanglement of the state is quantitatively an increasing function in $|r_{2}-s_{2}|$. According to the trace norm criterion [15], the entanglement of state ρ can be detected if $|t_{1}\left|>1-\right|t_{2}\left|-\right|t_{3}\left|=\right|t_{1}^{\prime }|$. However, based on our analysis, there are always local parameters $r_{2}$ and $s_{2}$ such that the state in Equation (19) is entangled if and only if the correlation parameters $t_{1}t_{3}\neq 0$. Further, we found that there are always some local parameters $r_{2}$ for any valid $s_{2}$ such that the state is entangled if and only if the correlation parameters $t_{1}t_{2}t_{3}<0$.

## Data Availability

Not applicable.
